# Genome-Wide Development of MicroRNA-Based SSR Markers in *Medicago truncatula* with Their Transferability Analysis and Utilization in Related Legume Species

**DOI:** 10.3390/ijms18112440

**Published:** 2017-11-18

**Authors:** Xueyang Min, Zhengshe Zhang, Yisong Liu, Xingyi Wei, Zhipeng Liu, Yanrong Wang, Wenxian Liu

**Affiliations:** 1 State Key Laboratory of Grassland Agro-ecosystems, Key Laboratory of Grassland Livestock Industry Innovation, Ministry of Agriculture, College of Pastoral Agricultural Science and Technology, Lanzhou University, Lanzhou 730020, China; minxy15@lzu.edu.cn (X.M.); zhangzs14@lzu.edu.cn (Z.Z.); weixy16@lzu.edu.cn (X.W.); lzp@lzu.edu.cn (Z.L.); yrwang@lzu.edu.cn (Y.W.); 2 China Telecom Gansu Wanwei Company, Lanzhou 730030, China; yisongliu2000@163.com

**Keywords:** *Medicago truncatula*, microRNA, microsatellites, genetic diversity, alfalfa

## Abstract

Microsatellite (simple sequence repeats, SSRs) marker is one of the most widely used markers in marker-assisted breeding. As one type of functional markers, MicroRNA-based SSR (miRNA-SSR) markers have been exploited mainly in animals, but the development and characterization of miRNA-SSR markers in plants are still limited. In the present study, miRNA-SSR markers for *Medicago truncatula* (*M. truncatula*) were developed and their cross-species transferability in six leguminous species was evaluated. A total of 169 primer pairs were successfully designed from 130 *M. truncatula* miRNA genes, the majority of which were mononucleotide repeats (70.41%), followed by dinucleotide repeats (14.20%), compound repeats (11.24%) and trinucleotide repeats (4.14%). Functional classification of SSR-containing miRNA genes showed that all targets could be grouped into three Gene Ontology (GO) categories: 17 in biological process, 11 in molecular function, and 14 in cellular component. The miRNA-SSR markers showed high transferability in other six leguminous species, ranged from 74.56% to 90.53%. Furthermore, 25 Mt*-*miRNA-SSR markers were used to evaluate polymorphisms in 20 alfalfa accessions, and the polymorphism information content (PIC) values ranged from 0.39 to 0.89 with an average of 0.71, the allele number per marker varied from 3 to 18 with an average of 7.88, indicating a high level of informativeness. The present study is the first time developed and characterized of *M. truncatula* miRNA-SSRs and demonstrated their utility in transferability, these novel markers will be valuable for genetic diversity analysis, marker-assisted selection and genotyping in leguminous species.

## 1. Introduction

Studying the genetic diversity and population structure of germplasm resources is not only important for understanding the genetic basis of traits, but also necessary for the discovery of new germplasm characteristics in order to develop and utilize germplasm resources for plant improvement [1,2,3,4]. Molecular markers have become an efficient way to identify polymorphisms among different genotypes or alleles at the DNA level, and played an increasing role in plant molecular breeding studies [2,5,6]. DNA-based markers can be divided into two types: one type is non-polymerase chain reaction (PCR)-based markers (RFLP, restriction fragment length polymorphism) and the other type includes PCR-based markers (RAPD, random amplified polymorphic DNA; AFLP, amplified fragment length polymorphism; SSR, simple sequence repeat; SNP, single nucleotide polymorphism; and ILP, intron length polymorphism). Within the second type, SSR marker is one of the most commonly used marker type with many genetic applications, such as map construction, comparative mapping, fingerprinting and genetic diversity, because of their good reproducibility, codominant nature, high information content, high abundance, robustness and single locus nature [7,8,9,10,11]. Furthermore, previous studies have documented that SSRs play pivotal roles in the regulation of chromatin organization, gene activity, and DNA metabolic processes [12]. The variation of repeat number in SSR loci which located in or linkage to functional genes have impacts on gene regulation, transcription, translation and protein function [2,3,7,9]. When SSRs are located in the intergenic region of the genome, such as expressed sequence tags (ESTs), transcriptome sequences, and miRNA genes, the SSR markers derived from these regions could be acted as “functional genetic markers” and widely used for marker assisted breeding and genomic selection [9,13].

miRNAs are a new class of small, endogenous and non-coding RNAs; they have been a significant avenue of research due to their critical role in development, differentiation, growth, metabolism and disease-resistance processes and in response to biotic and abiotic stresses through post-transcriptional regulation of gene expression in plants [14,15,16,17,18]. It is believed that miRNAs are generated from precursor RNA (pre-miRNA) with hairpin structures. Evolutionarily, some miRNA families are highly conserved through hundreds of millions of years in both plants and animals. Individual species also use species-specific miRNAs to regulate various developmental and biological processes [19,20]. Hence, miRNAs is useful to study genetic diversity among closely related genotypes. However, miRNA-SSR markers, as types of functional markers, have been used mainly in animals, and there have been very few reported uses in plants, except in the model rice plants, *Setaria italic*, *Nelumbo nucifera* and *Brassica* [12,21,22,23,24].

*M. truncatula* has been selected as a model plant to study functional genomics of legumes because of its self-fertile, small diploid genome, and high transformation efficiency [25]. With the rapid development of next-generation sequencing, the development of miRNA-SSR markers has become a relatively straightforward task by using a large number of deposited datasets. By now, a total of 1348 *M. truncatula* miRNAs have been identified, which provided an excellent opportunity to develop miRNA-based SSR markers at a genome-wide level [26]. Alfalfa (*Medicago sativa* L.) has long been taken as one of the most important planted forage crops worldwide because of its ability to provide reliable protein and minerals to animals [27,28,29,30]. As the sequence conservation between alfalfa and *M. truncatula* is relatively high, the estimation of maker collinearity between these two species is possible [31,32]. To date, a large number of SSR markers derived from *M. truncatula* have been developed and applied in legume crops, including alfalfa [33,34]. However, the development and usage of miRNA-SSR markers of *M. truncatula* have not been reported. This study reports the first genome-wide identification and development of SSR markers based on *M. truncatula* miRNAs; the markers’ potential for transferability and genetic diversity assessments in six leguminous also analysed.

## 2. Results and Discussion

### 2.1. The Frequency, Distribution and Characterization of miRNA-SSRs in the M. truncatula Genome

In the present study, a total of 1348 mature miRNA sequences from two public databases were used for the development of the miRNA-SSR markers. After removing the redundant sequences, 356 miRNAs remained and their corresponding pri-miRNA sequences were extracted from the *M. truncatula* genome and used for SSR site-mining (Table 1). These sequences represented approximately 407.9 kilobases (kb) and belonged to 248 *M. truncatula* miRNA families. A total of 189 SSRs were identified from 137 (38.48%) pri-miRNA sequences based on the *MISA* microsatellite search results, with a distribution frequency of one SSR locus per 0.5 kb, which was significantly lower than that of EST-derived SSRs (1.8 kb) [33] and transcription factor-derived SSRs (7.8 kb) in *M. truncatula* [34]. Analyses of SSR motifs in the SSR-containing pri-miRNA genes revealed that 46 (12.92%) pri-miRNA genes contained more than one SSR (Table 1). Of the 189 total SSRs, 168 (88.89%) contained simple repeat motifs, while 21 (11.11%) were compound motifs. These simple repeat motifs showed a diverse range of presentations with the mononucleotide motifs being the most abundant (133, 70.37%), followed by dinucleotide motifs (27, 14.29%) and trinucleotide motifs (8, 4.23%); no tetranucleotide, pentanucleotide or hexanucleotide repeats were found in any of the *M. truncatula* pri-miRNA sequences. The SSR length ranged from 10 to 64 bp, with 10 bp as the most frequently observed length (18.10%). Out of the 137 pri-miRNAs containing SSRs, the frequency of mononucleotides was the highest (0.77), present in 106 miRNA genes, and was followed by dinucleotides (0.17) occurring in 23 genes, while the frequency of trinucleotides was the lowest (0.05), present in only 7 miRNA genes (Figure S1A,B). The detailed numbers for these three kinds of repeat motifs are presented in Figure S1C.

In the present study, the percentage of miRNA genes possessing miRNA-SSRs was 38.48%, which is significantly higher than that reported in rice (15.5%) [22]. Out of the 189 miRNA-SSRs, chromosome 4 had the highest number of miRNA-SSRs, while chromosome 1 had the lowest. In addition, there were 38, 36, 27, and 26 miRNA-SSRs present on chromosomes 4, 3, 5, and 2, respectively (Figure S1D), composing 67.20% of the total miRNA-SSRs. These results indicate that the miRNA-SSRs identified in our study exhibited some location preference on these 4 chromosomes of the *M. truncatula* genome, and similar observations also have been found in rice [20].

### 2.2. Development of Mt-miRNA-SSR Markers

**Figure 1:**
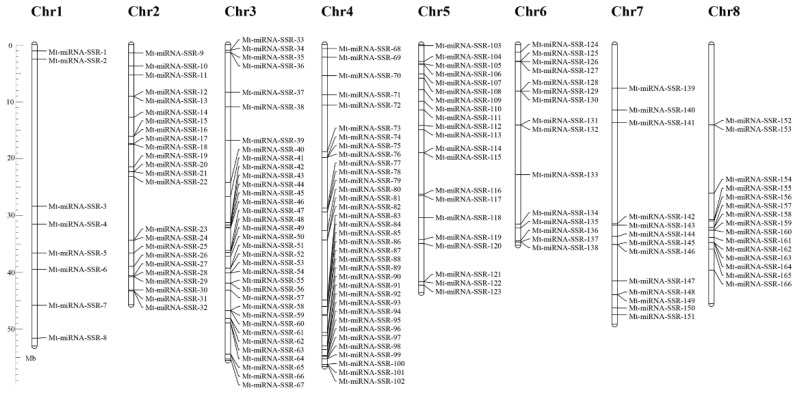
Physical map of miRNA-based markers. The chromosome numbers were shown at the top of each chromosome. The names on the right side of each chromosome correspond to the location of each miRNA-based marker.

Using the flanking regions of 189 SSR motifs, a total of 169 primer pairs were successfully designed from 130 (94.89%) *M. truncatula* miRNA genes; the remaining 20 SSR motifs failed for miRNA-SSR marker development because the flanking sequence of the SSR loci were too short or not match the criteria for primer design. The detailed information of these 169 successfully designed primer pairs are presented as supplementary data (Table S2). Of the 169 primer pairs, 119 (70.41%) were classified as mononucleotide repeats; 24 (14.20%), 19 (11.24%), and 7 (4.14%) were dinucleotide, compound, and trinucleotide repeats, respectively. Based on the BLAST analysis of these 169 miRNA-SSR markers against the *M. truncatula* genome, 166 markers could be physically mapped on all 8 chromosomes with an uneven ratio, and the other 3 markers (Mt-miRNA-SSR-167, 168, and 169) were located on the scaffold regions. Chromosomes 3 and 4 contained the largest number of markers (35, 21.08%), while the fewest mapped on chromosome 1 (8, 4.82%) (Figure 1).

Previously, 4636 EST-SSR markers [33] and 130 transcription factor gene-derived SSR markers had been developed from *M. truncatula* based on 3828 EST sequences and 176 transcription factor sequences, respectively [34]. To determine the novelty of the 169 Mt-miRNA-SSR markers developed in this study, the 130 SSR-containing pri-miRNA sequences were cross-referenced with the 3828 EST sequences and 176 transcription factor sequences, respectively. The BLASTN results found that only 5 out of 130 pri-miRNA sequences had significant similarity with 5 EST sequences. However, at the SSR loci level, only one SSR locus in pri-MIR396a was found to be similar to its homologous EST sequence, meaning that 168 of the 169 (99.41%) Mt-miRNA-SSR markers developed in our study are all novel.

### 2.3. Functional Classification of SSR-Containing miRNA Genes

To better understand the biological functions of the miRNAs used for Mt-miRNA-SSR marker development, the putative target genes of the miRNAs were identified using the psRNATarget program with the *M. truncatula* Mt4.0v1 spliced transcripts as the reference transcript library. Under the strict criteria described in Materials and Methods, a total of 105 putative targets were identified for 105 of 130 miRNAs. Out of the 105 predicted mRNA targets, the inhibition of 94 miRNA targets (89.52%) was caused by cleavage activity, while 11 targets (10.48%) were inhibited through translational repression (Table S3).

To further understand the functional features of these miRNA targets, the GO annotations of all the predicted targets were analysed. The most significant BLAST hits for each target across different species were obtained by BLASTP search and used for GO annotation. Based on the ontological definitions of the GO terms, all targets were classified into three GO categories: 17 biological process, 11 in molecular function, and 14 in cellular component (Figure S2). In the molecular function category (Figure S2A), predicated targets were primarily associated with binding, followed by catalytic activity. In the biological process category (Figure S2B), the majority of the targets were potentially involved in cellular processes, metabolic processes and single-organism processes. In the cellular component category (Figure S2C), 77 target genes were assigned to subcategory membrane, followed by membrane part, cell and cell part. Many of the predicated targets were annotated to be transcription factors (TFs), disease resistance proteins, and transmembrane proteins that are associated with multiple stress response processes (Table S3). Similarly, a statistical investigation of 65 functionally validated miRNAs in 9 major crops also indicated that nearly 70% of the target genes of these miRNAs are TFs and pathogen proteins, suggesting their important roles at the core of gene regulatory networks [35]. Up to now, the only set of trait specific miRNA derived SSR markers was developed in rice. With the help of these markers, both the tolerant and susceptible of rice genotypes could be differentiated, indicating their potential functions for the identification and characterization of salt sensitive rice genotypes [12]. Recently, a large number of miRNAs, which involved in various abiotic stresses, such as aluminium [36], salt [37], and drought [38], have been identified and studied in *M. truncatula*. Based on these stress-related miRNAs, the SSR markers developed in our study would be used as useful resources for the selection of desirable alleles and specific trait-related genotypes in *M. truncatula* and related legume species considering their high transferability as discussed later.

### 2.4. Transferability of Mt-mi-RNA-SSR Markers in Leguminous Species

All the Mt*-*mi-RNA-SSR markers across 8 chromosomes were selected for PCR amplification from the genomic DNA of *M. truncatula*. Among the 169 primer pairs, 157 were successfully amplified, and the remaining 12 failed to amplify PCR products at different annealing temperatures. Of the 157 successful primer pairs, 145 yielded amplification products of the expected size, while the PCR products generated by the other 12 primer pairs were smaller or larger than predicated. To further confirm the amplification of the expected Mt*-*mi-RNA-SSR markers, the PCR products of six primer pairs in *M. truncatula* were sequenced. The results showed that all the sequenced alleles from *M. truncatula* agreed with the corresponding locus that used for the marker development (Figure S3).

**Figure 2:**
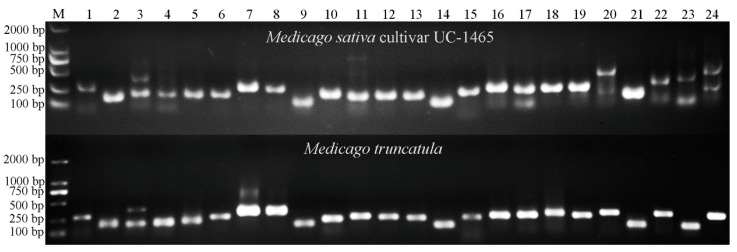
miRNA-SSR marker variations at the 24 Mt-miRNA-SSR loci of Medicago sativa cultivar UC-1465 and *M. truncatula*. The letter ‘M’ denotes the molecular markers, which are 100 bp, 250 bp, 500 bp, 750 bp, 1000 bp and 2000 bp (bottom to top).

As the most widely grown forage legume crop, alfalfa is particularly important worldwide due to its extensive ecological adaptability, high nutrition value, contribution to soil fertility, and potential as an energy crop [28,29]. However, limited information is available regarding the alfalfa genome, largely due to its complex genetics, large genome and intricate resistance mechanisms. Because of the close taxonomic relationship with the model legume species *M. truncatula*, the genetic marker colinearity between alfalfa and *M. truncatula* could be estimated and then further used for genetic diversity analysis in alfalfa. To assess the cross-species transferability, all of the Mt*-*mi-RNA-SSR markers developed in this study were tested in alfalfa, using *M. truncatula* as a positive control (Figure 2). One hundred twenty-six (74.56%) of the 169 assayed Mt*-*mi-RNA-SSR markers provided consistent amplification in alfalfa (Table 2). This result agrees with the previous study that high transferability possessed by the microsatellite markers developed from *M. truncatula* in alfalfa (85.21%) [34]. We further analyzed the transferability of these 169 Mt-mi-RNA-SSR markers in other five leguminous species, and high transferability also has been identified, such as 77.51% in *Glycine max*, 89.35% in *Vicia sativa*, 88.76% in *Melilotus*, 90.53% in *Lotus corniculatus*, and 89.35 in *Sophora alopecuroides* (Table 2). The high transferability of Mt*-*mi-RNA-SSR markers in these leguminous species indicates that the regions in the mi-RNA genes flanking the microsatellites are highly conserved [5], which will render these markers useful in the future identification and isolation of genes governing important agronomic traits.

### 2.5. Genetic Diversity Analysis of 20 Alfalfa Accessions

In order to test the application potential of the Mt-mi-RNA-SSR markers in genetic diversity and germplasm evaluation in alfalfa, the 126 Mt-mi-RNA-SSR transferable markers in alfalfa were selected and analyzed in 20 alfalfa accessions (Table S1). The results showed that all 126 primer pairs had clear and reproducible amplification and 25 were polymorphic across the 20 alfalfa accessions (Table 3). There were a total of 197 alleles detected from these 25 polymorphic Mt-mi-RNA-SSR markers, and 143 of these alleles were polymorphic. The number of alleles generated per primer pair varied from 3 (Mt-miRNA-SSR-10) to 18 (Mt-miRNA-SSR-37) with an average of 7.88. The Mt-miRNA-SSR-37 and Mt-miRNA-SSR-63 (0.89) had the highest PIC value, while the lowest PIC value was observed for Mt-miRNA-SSR-102 (0.39), and the average PIC value was 0.71 (Table 3). Previous research indicated that markers were referred as informative when their PIC values greater than 0.5 and markers with PIC values greater than 0.7 were suitable for genetic mapping [39]. In the present study, 16 and 24 Mt-miRNA-SSR markers had PIC values greater than 0.7 and 0.5, respectively, which indicates the potential for these markers in genetic diversity and genetic mapping analyses.

**Figure 3:**
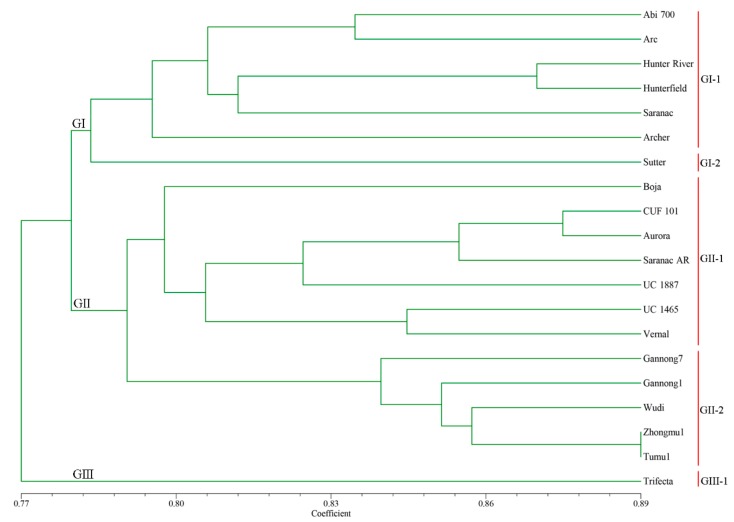
The dendrogram of 20 alfalfa accessions based on UPGMA analysis using 25 polymorphic Mt-miRNA-SSR markers.

The genetic relationships of 20 alfalfa accessions were determined by UPGMA cluster analysis based on the 25 polymorphic Mt-miRNA-SSR amplification results. The 20 alfalfa accessions could be grouped into three main groups (GI, GII and GIII) and five subgroups (GI-1, GI-2, GII-1, GII-1 and GIII) based on the cluster results (Figure 3). There were 7 accessions in group G1, which were divided into two subgroups. Subgroup GI-1 included 6 accessions collected from the United States, Australia and Mexico; subgroup GI-2 only contained one accession (Sutter) collected from the United States. The G2 group was also divided into two subgroups. Subgroup GII-1 included 7 accessions collected from the United States, Guatemala and Poland, and subgroup GII-2 contained 5 accessions all collected from China. The last group GIII only contained one accession (Trifecta) and was collected from the United States. Although all indigenous accessions clustered into a single subgroup (GII-2), the association between the geographical distribution and clustering pattern among the 15 exotic accessions was less significant, which may due to the number of markers was not enough to cover the large genome of alfalfa or fewer accessions from different geographical locations were used in this study. Previous studies have also reported similar results [40,41]. Furthermore, the *M. sativa notho. varia* cultivars were not formed as a separate cluster but scattered among the other 11 (GII) *M. sativa* ssp. *sativa* cultivars/landrace; this might be explained by the recurrent breeding methods involving multiple hybridizations and selection activities between *M. sativa Martin* and *M. sativa* ssp. *sativa* material in alfalfa. Nevertheless, the value of the novel Mt-miRNA-SSR markers developed in this research was emphasized by our results, and these markers may be useful for modern alfalfa breeding programmes and genetic diversity evaluation in alfalfa genotypes.

## 3. Materials and Methods

### 3.1. Plant Material and DNA Isolation

The *M. truncatula* (A17), alfalfa (cultivar UC-1465), *Glycine max* (cultivar Dongdou 641), *Vicia sativa* (cultivar Lanjian 3), *Melilotus* (cultivar Norgold), *Lotus corniculatus* (cultivar Mirabal) and *Sophora alopecuroides* (wild material) were used to examine the transferability of Mt-miRNA-SSR markers developed in this study. Genomic DNA was extracted from the leaves of greenhouse plants using a CTAB protocol as described previously [34]. Twenty alfalfa accessions were obtained from the United States Department of Agriculture National Plant Germplasm System (NPGS, http://www.ars-grin.gov/npgs/) and the National Animal Husbandry & Veterinary Service of the Ministry of Agriculture (MOA), State Grass Germplasm Resources and used for genetic diversity analysis (Table S1). For each accession, young leaves of 40 individual field plants were collected separately and bulked as one sample for genomic DNA isolation as described above. The DNA quality was determined using 1% agarose gel, and DNA quantity was determined using a NanoDrop ND1000 instrument (Thermo Scientific, Waltham, MA, USA).

### 3.2. Identification of SSRs and Primer Design

A total of 1348 miRNAs of *M. truncatula* were downloaded from PMDR [26] and miRBase ver 19 [42]. To avoid sequence redundancy, the similar sequences were removed using CD-HIT Suite (identity cut-off = 0.9) [43], and then the remain pre-miRNA sequence of non-redundant mature miRNAs were extracted and used as queries for a stand-alone BLASTN (Basic Local Alignment Search Tool, http://blast.ncbi.nlm.nih.gov) search against the *M. truncatula* genome (version Mt4.0v1, http://www.jcvi.org/medicago/index.php). When the pre-miRNA sequences 100% matched (*E* value = 10^−10^) to the *M. truncatula* genome sequence, their corresponding hit sequences along with 500 bp from the 5′ and 3′ flanking sites were extracted. The 1000 base pairs (bp) sequences along with the respective pre-miRNA sequence were then referred as the primary miRNA (pri-miRNA) and used for identification and localization of SSRs with a Perl 5 script (*MISA*, MIcroSAtellite identification tool; http://pgrc.ipk-gatersleben.de/misa/). Repeats with a minimum of 10 and 6 were defined for the mono- and dinucleotides, respectively, and repeats of 5 were defined for tri-, tetra-, penta-, and hexanucleotides. For the compound microsatellites type, 100 bp length was set as the maximum interruption between 2 SSRs. Flanking primers to the SSRs were designed by Primer3 software with the help of Perl 5 interface modules.

### 3.3. Prediction of miRNA Targets and Functional Gene Ontology (GO) Analysis

The psRNATarget Server was used for the identification of the putative mRNA targets of the miRNAs with default parameters [44]. The miRNA sequences corresponding to the pri-miRNAs were used as queries and the *M. truncatula* Mt4.0v2 spliced transcripts were used as the reference transcript library for the target search. To further understand the function of miRNA targets, GO classification was applied. BLASTP (*E* value = 10^−6^) was employed to perform a homology search against the NCBI non-redundant (Nr) protein database to obtain the most significant BLAST hits, and then the GI list transformed into GO numbers by TBtools (https://github.com/CJ-Chen/TBtools).

### 3.4. PCR Amplification

A final volume of 20 µL mix containing 50 ng template DNA, 2.0 mM MgCl_2_, 1× PCR buffer, 2.5 mM dNTPs, 0.8 unit *Taq* polymerase (TaKaRa, Dalian, China), and 4 µM each primer was used for PCR amplification. The PCR reaction conditions were the following: a hot-start at 94 °C for 4 min, followed by 35 cycles at 94 °C for 30 s, 60 °C for 35 s, and 72 °C for 1 min, with a final elongation step at 72 °C for 5 min. The PCR products were then subjected to electrophoresis on 6.0% polyacrylamide gels, and the banding patterns were visualized using silver staining [34]. Three independent PCR amplifications were performed for each primer.

### 3.5. Genetic Diversity Analysis

The SSR profiles (alleles) were scored for absent (0) or present (1) of the corresponding band among different alfalfa accessions. Only specific bands that could be unambiguously scored were used for statistical analysis. PIC-CALC 0.6 was used to calculate Polymorphism information content (PIC) [40]. A dendrogram was generated based on the genetic identity matrix using the unweighted pair-group mean algorithm (UPGMA) of NTSYSpc software (version 2.02K, Applied Biostatistics, Inc., New York, NY, USA).

## Figures and Tables

**Table 1:** Summary of SSR search results.

Search Items	Numbers
Total number of pri_miRNA sequence examined	356
Total number of identified SSRs	189
Number of SSR containing sequences	137
Number of pri-miRNA containing more than 1 SSR	46
Number of SSRs present in compound formation	21
Repeat type	
Mononucleotide	133
Dinucleotide	27
Trinucleotide	8
Total length of sequences searched (kb)	407.9
Frequency of SSRs	One per 0.5 kb

**Table 2:** Transferability of Mt-miRNA-SSR markers in leguminous species.

Species	Transferability
*Medicago truncatula*	157 (92.90%)
*Medicago sativa*	126 (74.56%)
*Glycine max*	131 (77.51%)
*Vicia sativa*	151 (89.35%)
*Melilotus*	150 (88.76%)
*Lotus corniculatus*	153 (90.53%)
*Sophora alopecuroides*	151 (89.35%)

**Table 3:** Details of the 25 polymorphic Mt-miRNA-SSR markers with their genetic parameter values.

No.	Primer Name	Primer Sequence (5′–3′)	No. of Alleles	He	PIC Value
1	Mt-miRNA-SSR-7	F: CGCAACCAACATAGAAGCAA	11	0.84	0.82
		R: CGCGGTCTTATTAGGGATCA			
2	Mt-miRNA-SSR-10	F: GCATCGCCGTTATTAACAAAA	3	0.56	0.47
		R: CGGCTTCATACACAGGGAAT			
3	Mt-miRNA-SSR-26	F: TTGCAAACCAAACACACACA	5	0.78	0.74
		R: GCGACATACAATTTGGGCTT			
4	Mt-miRNA-SSR-28	F: CAAACGTTTTCTCAATTTCTAATCG	7	0.71	0.66
		R: CCAAGGTTGTTCCAAGGTGT			
5	Mt-miRNA-SSR-29	F: AGCCTCTCATTTAATTGGTGC	4	0.69	0.62
		R: GCAGGTGCAAATGCAGATAA			
6	Mt-miRNA-SSR-37	F: TCCTTTGCTCTTCCAACTCTTT	18	0.90	0.89
		R: CCCCCTTTGTTAGCAGATGA			
7	Mt-miRNA-SSR-38	F: AATGTATGGAGAGGATGAGCTTT	6	0.68	0.65
		R: AACCAGATTACCTTCATCATTCG			
8	Mt-miRNA-SSR-41	F: TGGTTCAGAAACGGTTAGGG	5	0.76	0.72
		R: CAGAAAGGTCCAGAAGCCAA			
9	Mt-miRNA-SSR-45	F: GTGAAGCAATGGTGCCTTTT	10	0.75	0.71
		R: TCACGGCTCAAAGGTATGTG			
10	Mt-miRNA-SSR-47	F: TCAATCAGAAAAATTGCACCC	13	0.87	0.86
		R: AAAGTTTTTGTTGGGAAAGATGA			
11	Mt-miRNA-SSR-52	F: ATTTTGTGTGCCATCGTGAA	14	0.85	0.83
		R: GGGACCGGTTATCTTTTGGT			
12	Mt-miRNA-SSR-63	F: GCCATGTTTTGCATGACTGT	13	0.90	0.89
		R: TGCAGGTCCAAATTCAACAA			
13	Mt-miRNA-SSR-87	F: TGAAATGCCTTTTTCTTCCC	7	0.83	0.81
		R: TTCCCAAACACCATCATCAA			
14	Mt-miRNA-SSR-99	F: GAGGACGGATCAATAGGCAA	4	0.69	0.62
		R: TGTCTGGAAAGTGCTTCACAA			
15	Mt-miRNA-SSR-102	F: CCACGATGCTACACACGTTC	4	0.44	0.39
		R: CTTCCACGTCCAGACCAACT			
16	Mt-miRNA-SSR-125	F: TAGCCCTGCCAGCCTATTTA	15	0.81	0.79
		R: AAGGTGTCATCTCTCCTGCG			
17	Mt-miRNA-SSR-126	F: TTCTCCAGCAGTGCTATTCTGA	7	0.83	0.81
		R: TGCTGTTCCTTTGTTTTCAATG			
18	Mt-miRNA-SSR-130	F: TCCATGTTTTTGGCATCAGA	4	0.60	0.52
		R: AATTGGGGAAATAAGGGGTG			
19	Mt-miRNA-SSR-142	F: CCAAAAAGATTTGGCCCTTT	6	0.81	0.78
		F: GCATGGTTGTCCCTTGCTAT			
20	Mt-miRNA-SSR-144	R: TGCTTGTTCAATTTCGAATG	4	0.69	0.62
		F: CTTAAGTTACCTGTCCGGCG			
21	Mt-miRNA-SSR-148	R: TGCTCATGTTGATTCCCAGA	5	0.58	0.50
		F: AGCATTAGTTGTCATGCCCC			
22	Mt-miRNA-SSR-151	R: AAACATGTGGGGTTTGGTGT	10	0.89	0.88
		F: AGCCAAGTTTGGACCATCAG			
23	Mt-miRNA-SSR-163	R: GGGGAGGAAAGGTTGAATTT	12	0.87	0.86
		F: GCTGCAGTTAACTACCGAGGA			
24	Mt-miRNA-SSR-164	R: GGGGAGGAAAGGTTGAATTT	6	0.78	0.74
		F: GCTGCAGTTAACTACCGAGGA			
25	Mt-miRNA-SSR-166	R: GCACCATTAGTGTGGTGTGAG	4	0.70	0.64
		F: GCCAACATTCCCCTCAAATA			
		Average	7.88	0.75	0.71

## Supplementary Materials

Supplementary materials can be found at www.mdpi.com/1422-0067/18/11/2440/s1.
