# Supplementary material for: Genome-Wide Development of MicroRNA-Based SSR Markers in Medicago truncatula with Their Transferability Analysis and Utilization in Related Legume Species
Source: Int J Mol Sci. 2017 Nov 18;18(11):2440. doi: 10.3390/ijms18112440 (PMC5713407; doi:10.3390/ijms18112440)
Supplement: Supplementary file 1 [file ijms-18-02440-s001.zip › Supplementary Files/Table S1.docx]

**Table S1.** List of 20 alfalfa accessions used for genetic diversity analysis in this study.

| **No.** | **Name** | **Species** | **Type** | **Country of origin** |
| --- | --- | --- | --- | --- |
| 1 | Abi 700 | *M. sativa ssp. sativa* | Cultivar | United States |
| 2 | Arc | *M. sativa ssp. sativa* | Cultivar | United States |
| 3 | Archer | *M. sativa ssp. sativa* | Cultivar | United States |
| 4 | Boja | *M. sativa notho. varia* | Cultivar | Poland |
| 5 | CUF 101 | *M. sativa ssp. sativa* | Cultivar | United States |
| 6 | Hunter River | *M. sativa ssp. sativa* | Cultivar | Mexico |
| 7 | Hunterfield | *M. sativa ssp. sativa* | Cultivar | Austria |
| 8 | Aurora | *M. sativa ssp. sativa* | Cultivar | Guatemala |
| 9 | Saranac | *M. sativa ssp. sativa* | Cultivar | United States |
| 10 | Saranac AR | *M. sativa ssp. sativa* | Cultivar | United States |
| 11 | Sutter | *M. sativa ssp. sativa* | Cultivar | United States |
| 12 | Trifecta | *M. sativa ssp. sativa* | Cultivar | Austria |
| 13 | UC-1465 | *M. sativa ssp. sativa* | Cultivar | United States |
| 14 | UC-1887 | *M. sativa ssp. sativa* | Cultivar | United States |
| 15 | Vernal | *M. sativa ssp. sativa* | Cultivar | United States |
| 16 | Gannong7 | *M. sativa ssp. sativa* | Cultivar | China |
| 17 | Gannong1 | *M. sativa ssp. sativa* | Cultivar | China |
| 18 | Wudi | *M. sativa ssp. sativa* | Land race | China |
| 19 | Zhongmu1 | *M. sativa ssp. sativa* | Cultivar | China |
| 20 | Tumu1 | *M. sativa Martin* | Cultivar | China |
