# Supplementary figures and images for: Genome-Wide Development of MicroRNA-Based SSR Markers in Medicago truncatula with Their Transferability Analysis and Utilization in Related Legume Species
Source: Int J Mol Sci. 2017 Nov 18;18(11):2440. doi: 10.3390/ijms18112440 (PMC5713407; doi:10.3390/ijms18112440)

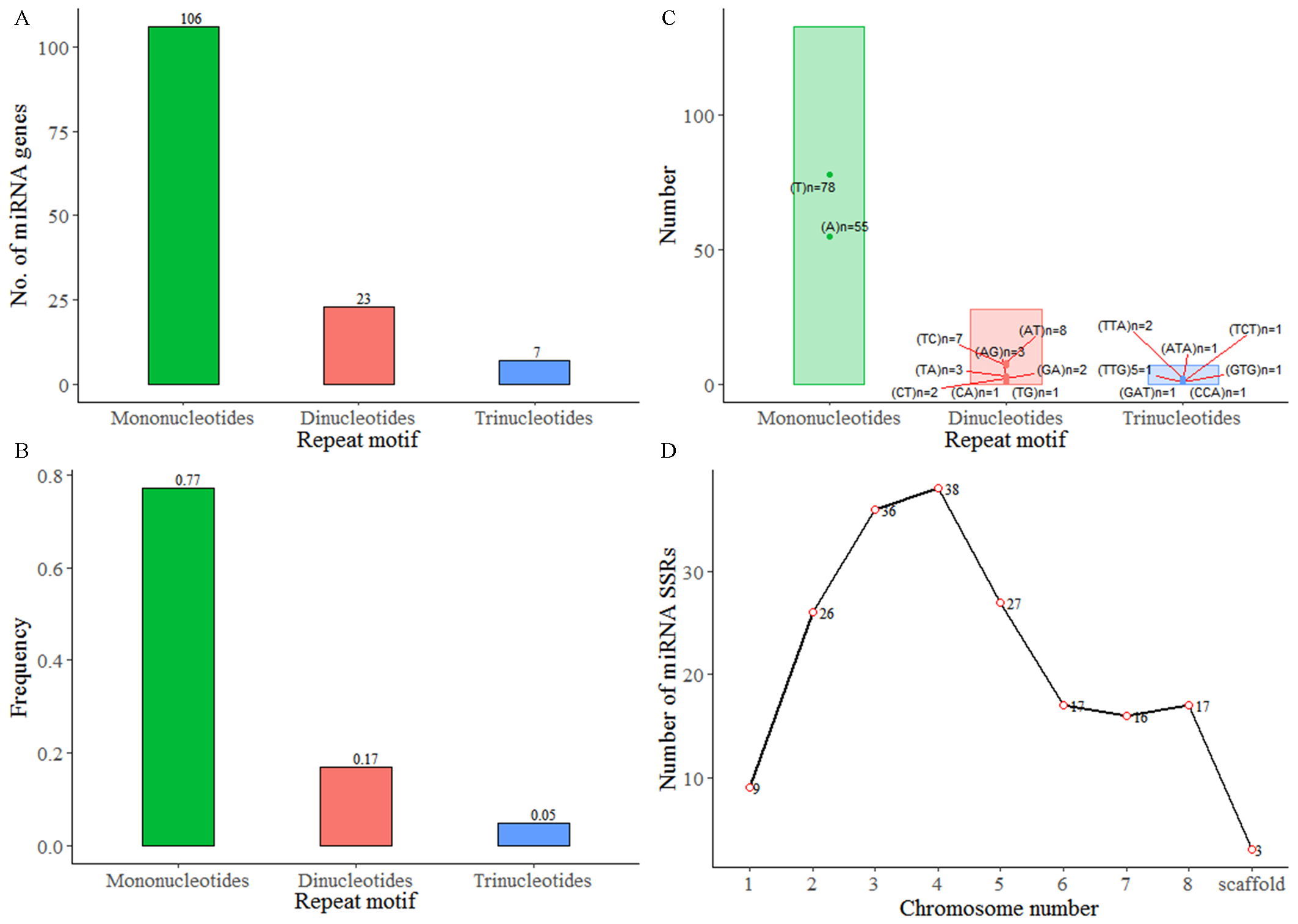

Supplement: Supplementary file 1 [file ijms-18-02440-s001.zip › Supplementary Files/Figure S1.tif]

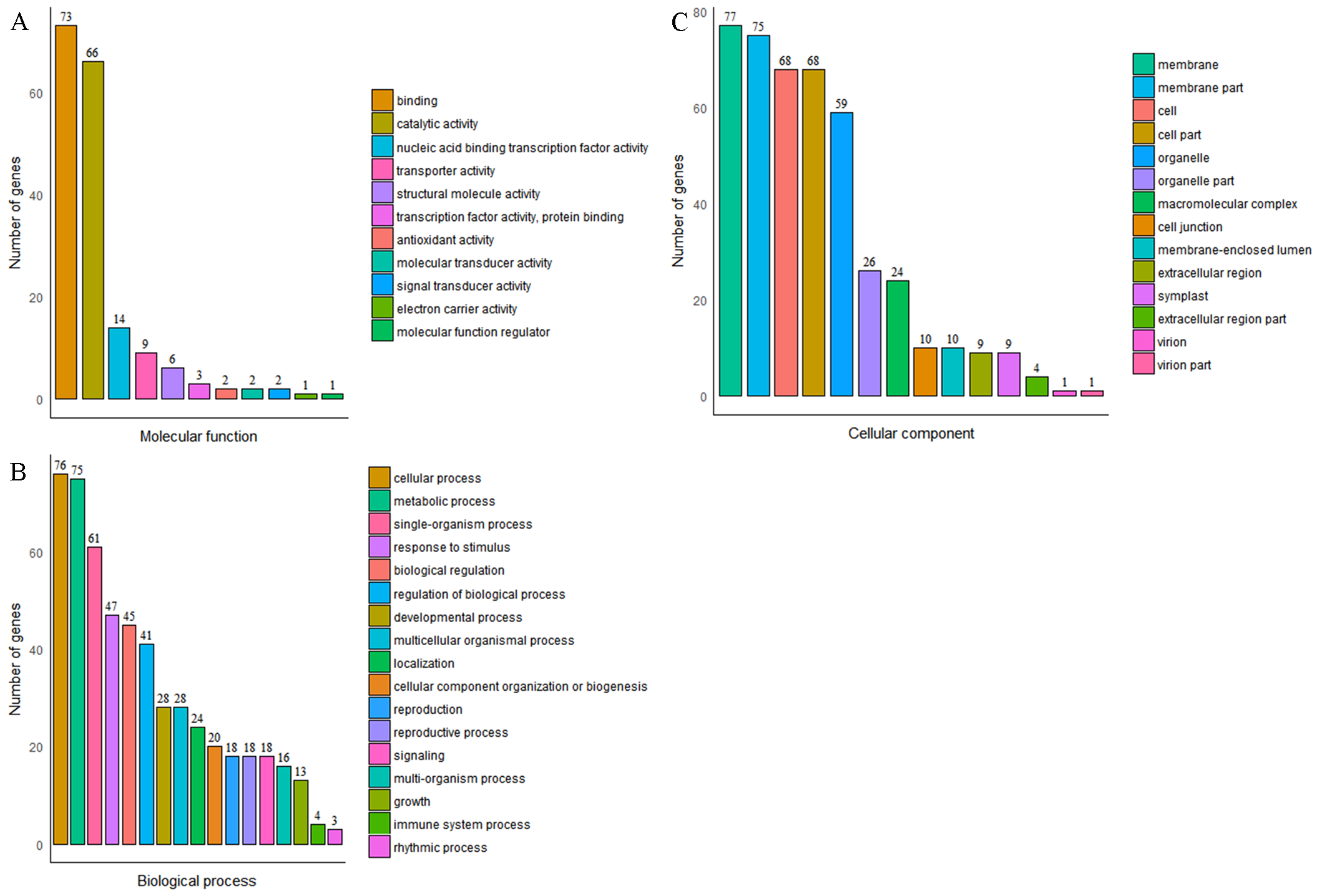

Supplement: Supplementary file 1 [file ijms-18-02440-s001.zip › Supplementary Files/Figure S2.tif]

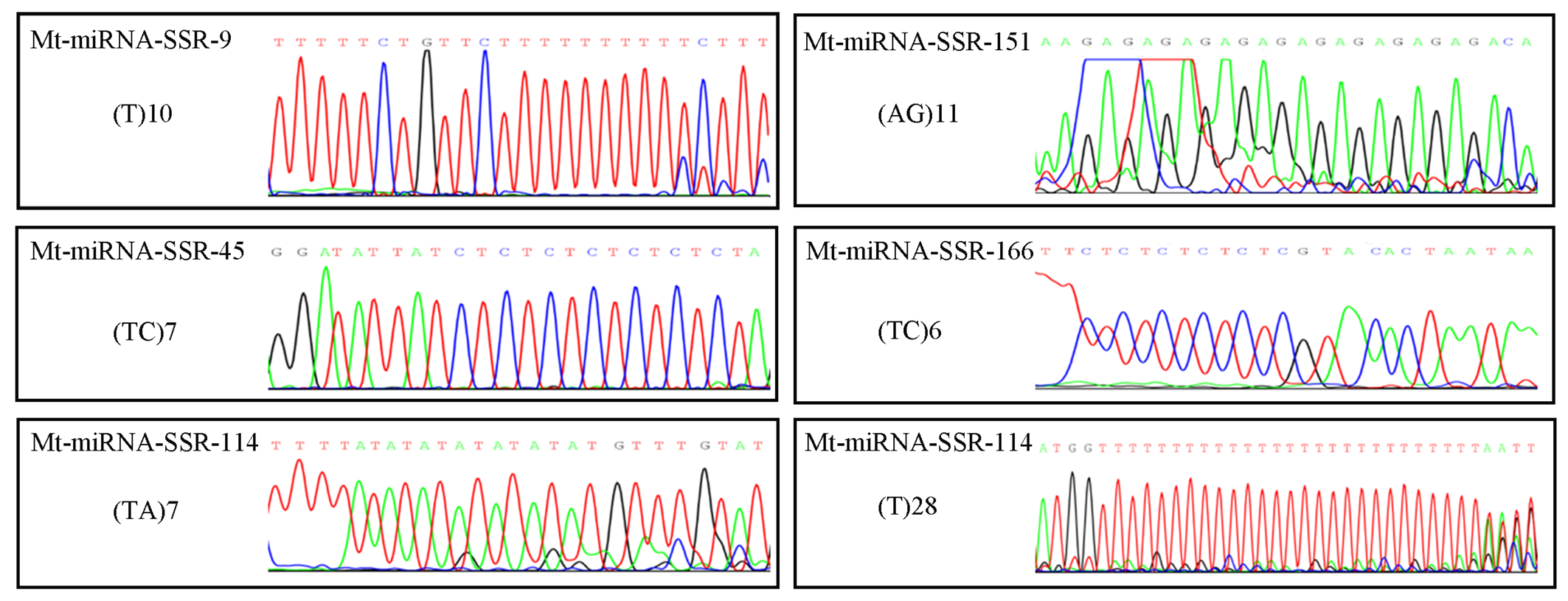

Supplement: Supplementary file 1 [file ijms-18-02440-s001.zip › Supplementary Files/Figure S3.tif]
