# Supplementary material for: Genome-Wide Development of MicroRNA-Based SSR Markers in Medicago truncatula with Their Transferability Analysis and Utilization in Related Legume Species
Source: Int J Mol Sci. 2017 Nov 18;18(11):2440. doi: 10.3390/ijms18112440 (PMC5713407; doi:10.3390/ijms18112440)
Supplement: Supplementary file 1 [file ijms-18-02440-s001.zip › Supplementary Files/Supplementary Figure Legends.doc]

**Genome-wide Development of microRNA-based Microsatellite Markers of *Medicago truncatula* and Transferability Analysis for Utilization in Alfalfa**

**Xueyang Min****^1^, Zhengshe Zhang^1^, Yisong Liu^2^, Xingyi Wei^1^, Zhipeng Liu^1^, Yanrong Wang^1^ and Wenxian Liu^1^***

**Figure legends**

**Figure S1.** The frequency, distribution and characterization of miRNA-SSRs in M. truncatula genome. **(A)** The number of repeat motifs. **(B)** The frequency of repeat motifs. **(C)** The detailed numbers of motif type for three kind of repeat motifs. **(D)** Distribution of miRNA-SSRs on each of the 8 chromosomes of *M. truncatula.*

**Figure S2.** Functional classification of SSR containing miRNA genes in *M. truncatula*.

**Figure S3.** Comparative electropherogram analysis of five miRNA-SSRs loci (Mt-miRNA-SSR-9, -45, -114-, 151, -166) in *M. truncatula*.
